# The relationship between mean platelet volume lymphocyte ratio and collateral circulation in patients with chronic total coronary occlusion

**DOI:** 10.3389/fcvm.2022.1008212

**Published:** 2022-10-28

**Authors:** Ming-Hui Niu, Peng-Hui Liu, Ze-Hua Liu, Jun-Wei Zhu, Rong Guo, Fei He

**Affiliations:** ^1^Department of Cardiology, The First Affiliated Hospital of Zhengzhou University, Zhengzhou, China; ^2^Department of Hematology, The First Affiliated Hospital of Zhengzhou University, Zhengzhou, China

**Keywords:** chronic total occlusion, collateral circulation, mean platelet volume, lymphocyte, inflammation

## Abstract

**Objective:**

To correlate mean platelet volume lymphocyte ratio (MPVLR) and coronary collateral circulation (CCC) in patients with chronic total occlusion (CTO).

**Materials and methods:**

A total of 643 patients who were hospitalized at a single large academic medical center from January 2020 to October 2021 and had CTO lesions in at least one major coronary artery confirmed by coronary angiography were retrospectively analyzed. Patients were divided according to the Rentrop criteria into poorly formed CCC (Rentrop grade 0–1, *n* = 235) and well-formed CCC (Rentrop grade 2–3, *n* = 408) groups. Mean platelet volume lymphocyte ratio (MPVLR) was calculated from routine laboratory data (MPV divided by lymphocyte count). The clinical data of the two groups were compared, and relationships between MPVLR and CCC formation were analyzed.

**Results:**

The MPVLR of patients with poorly formed CCC was significantly higher than that of patients with well-formed CCC (7.82 ± 3.80 vs. 4.84 ± 1.42, *P* < 0.01). The prevalence of diabetes mellitus and C-reactive protein levels were significantly higher in the poor CCC group than in the good CCC group (*P* < 0.01), while the proportions of patients with CTO or multivessel lesions in the right coronary artery were significantly lower in the poor CCC group than in the good CCC group (*P* < 0.01). Multivariate logistic regression analysis identified MPVLR (OR: 2.101, 95% CI: 1.840–2.399, *P* < 0.01), C-reactive protein level (OR: 1.036, 95% CI: 1.008–1.064, *P* < 0.05), a history of diabetes mellitus (OR: 2.355, 95% CI: 1.532–3.621, *P* < 0.01), and right coronary CTO ratio (OR: 0.313, 95% CI: 0.202–0.485, *P* < 0.01) as independent risk factors for CCC formation. The area under the ROC curve of MPVLR for predicting poorly formed CCC was 0.82 (95% CI: 0.784–0.855, *P* < 0.01), the best cut-off point was 6.02 and the sensitivity and specificity of MPVLR for predicting poorly formed CCC were 72.3 and 82.4%, respectively.

**Conclusion:**

In patients with coronary CTO, MPVLR was negatively correlated with CCC and a high MPVLR level was an independent predictor of poorly formed CCC.

## Introduction

Chronic total occlusion (CTO) is a serious and complex coronary artery disease. The formation of good coronary collateral circulation is particularly important for those CTO patients who would be difficult to revascularize (1). Good collateral circulation can increase myocardial perfusion to the ischemic area, improve cardiac systolic function, reduce future cardiovascular events (2–5). Methods for evaluating collateral circulation currently include coronary angiography and myocardial perfusion imaging, etc. These procedures are relatively complex and expensive. It is therefore necessary to find a simple and feasible method for evaluating and predicting the formation of CCC.

Inflammatory response plays a key role in all stages of atherosclerosis and collateral angiogenesis (6). Mean platelet volume to lymphocyte ratio (MPVLR) is a newly discovered inflammatory index that has been studied in a variety of diseases such as strokes and hepatitis (7, 8). Mean platelet volume (MPV) is an indicator of platelet function and activation (9). Hadadi et al. (10) showed that MPV and platelet-to-lymphocyte ratio can predict the presence of CTO in patients presenting with ST-segment elevation myocardial infarction (STEMI). Hudzik et al. (11) showed that acute coronary syndrome patients with an elevated MPVLR had a higher coronary thrombosis burden, and that increased MPVLR was an independent risk factor for early and late death in patients with STEMI. Kurtul et al. (12) found that MPVLR was an independent predictor of no-reflow after percutaneous coronary intervention in patients with STEMI.

The formation of coronary collateral circulation is closely related to the inflammatory response. MPVLR as a comprehensive indicator reflecting platelet function and inflammatory cells may be related to the formation of coronary collateral circulation. Ornek et al. (13) preliminarily studied the correlation between MPVLR and coronary collateral circulation in patients with stable angina pectoris. There are no relevant reports of MPVLR in China. These prior studies also had small sample sizes, and therefore require further confirmation. This study therefore aimed to investigate the viability of MPVLR as a new and simple predictor of the semi quantity of compensatory collateral circulation in CTO patients.

## Materials and methods

### Study population

A total of 643 hospitalized patients who underwent coronary angiography at the Department of Cardiology of the First Affiliated Hospital of Zhengzhou University from January 2020 to October 2021 were enrolled. CTO lesions were confirmed in at least one major coronary artery (left anterior descending artery, LAD; left circumflex artery, LCX; and right coronary artery, RCA) *via* coronary angiography.

The diagnosis of CTO was based on the guidelines established by the 2019 European CTO Club consensus document for recanalization of CTO, which were the complete occlusion of a coronary artery for more than 3 months with TIMI grade 0 distal blood flow (14). Exclusion criteria were: (1) an acute myocardial infarction within the past 3 months; (2) percutaneous coronary intervention and/or coronary artery bypass grafting within the previous 3 months; (3) coronary myocardial bridging and/or congenital coronary malformation; (4) severe heart failure (left ventricular ejection fraction < 30%), severe heart valve disease or cardiomyopathy; (5) severe liver or renal [estimated glomerular filtration rate (eGFR) < 30 ml/min/1.73 m^2^] impairment; and (6) severe infectious disease, systemic inflammatory diseases, malignancy or hematological diseases. The study protocol was approved by the ethics committee of the First Affiliated Hospital of Zhengzhou University, and written informed consent was obtained from all patients.

### Laboratory analysis

#### Clinical data collection

For cases that met the inclusion criteria, the patient’s name, gender, age, cardiovascular risk factors (hypertension, diabetes, smoking history, family history), medication history (aspirin, clopidogrel, statins, ACEI/ARB, *β*-blockers, calcium channel blockers), and coronary angiography data were collected. Left ventricular ejection fraction (LVEF) measured by echocardiography and the systolic blood pressure (SBP) and diastolic blood pressure (DBP) of the right upper arm at the time of admission were collected. Venous blood samples were routinely collected on the morning of the day after admission after at least 8 h after fasting. Collection included a routine basic complete blood count (white blood cell count, red blood cell count, platelet count, neutrophil, lymphocyte absolute value, and absolute value average platelet volume), total cholesterol, low-density lipoprotein cholesterol, high-density lipoprotein cholesterol, triglycerides, c-reactive protein (CRP), and creatinine. MPVLR was calculated by dividing the MPV by the lymphocyte count.

Hypertension was defined according to the 2018 ESC/ESH Hypertension guidelines (15) as an office systolic blood pressure ≥ 140 mmHg and/or diastolic blood pressure ≥ 90 mmHg, 24-h ambulatory blood pressure monitoring ≥ 130/80 mmHg, or home blood pressure monitoring ≥ 135/85 mmHg. The diagnostic criteria for diabetes were based on the International Diabetes Federation’s 2012 global guidelines for type 2 diabetes (16): a fasting blood glucose ≥ 7 mmol/L 2 h after glucose loading, a random blood glucose ≥ 11.1 mmol/L or a glycated hemoglobin level ≥ 6.5%. Smoking history was defined as continuous or cumulative smoking for 6 months or more over a lifetime. Familial CHD was described as being younger than 55 years of age in men and older than 65 years of age in women at the time of diagnosis with a first-degree relative with a history of CHD or sudden cardiac death.

#### Coronary angiography methods

All patients underwent coronary angiography *via* the radial or femoral artery using the standard Judkins technique. Results were evaluated by two experienced interventional cardiologists, and CCC was graded according to the Cohen-Rentrop criteria (17): Grade 0: no CCC formation (no contrast agent filling at the distal end of the occlusion); Grade 1: collateral perfusion beside the occluded vessels, but the vascular development was very weak; Grade 2: the distal collateral branches of the occluded vessels were developed at a lower density and slower filling rate than the feeding vessels; and Grade 3: the occluded distal vessels were fully developed, with the same density and feeding collateral branches and a faster filling rate. In patients with multiple coronary lesions or when multiple coronary collateral branches were present, the highest Rentrop grade was selected. Polyvascular disease was defined as the presence of lesions in two or more large epicardial arteries. Patients were further divided into poorly formed CCC (Rentrop grade 0–1) and well-formed CCC (Rentrop grade 2–3) groups.

### Statistical analysis

SPSS 22.0 (IBM, Armonk, NY, USA) was used for statistical analysis. The Kolmogorov-Smirnov test was used to test the normality of the measurement data. Normally distributed data was expressed as mean ± standard deviation and independent sample *t*-tests were used for comparisons between these groups. Non-normally distributed data were expressed as median and interquartile range, and Mann–Whitney *U* tests were used. Enumeration data were expressed as frequency (rate), the *χ*^2^ test was used for comparisons between groups, and a one-way analysis of variance (ANOVA) was performed to compare Rentrop grade categories. The Spearman test was performed to describe correlations of research indicators with the Rentrop grade. Possible influencing factors for CCC formation were analyzed using logistic univariate analysis and multivariate regression analysis. Variables with *P* < 0.05 after univariate analysis were included in the multivariate regression analysis. A receiver operating characteristic (ROC) curve was used to describe the predictive value of these factors. MedCalc version 20.111 (MedCalc software Ltd., Ostend, Belgium) was used to compare the area under the ROC curves of two indicators. *P* < 0.05 was considered statistically significant.

## Results

### Baseline comparison of patients with poor and good collateral circulation

A total of 643 patients met our inclusion and exclusion criteria, including 235 patients in the poor CCC group and 408 patients in the good CCC group. There were no significant differences in age, gender, body mass index (BMI), history of hypertension, smoking, drinking, family history of coronary heart disease, systolic and diastolic blood pressure at admission, left ventricular ejection fraction and medication for coronary heart disease between the two groups (all *P* > 0.05). The proportion of patients with diabetes in the poor CCC group was significantly higher than that of the good CCC group (45.5% vs. 32.1%, *P* < 0.05, Table 1).

**TABLE 1:** Comparison of baseline data between the two groups.

Variables	Poor CCC (*n* = 235)	Good CCC (*n* = 408)	*P*-value
^&^Age (years)	60.9 ± 10.3	59.1 ± 11.3	0.051^a^
^&^BMI (kg/m^2^)	24.7 ± 2.9	25.1 ± 3.0	0.133^a^
^†^Men (*n*, %)	175 (74.5)	318 (77.9)	0.316^b^
^†^Smoking history (*n*, %)	88 (37.4)	178 (43.6)	0.125^b^
^†^History of drinking (*n*, %)	58 (24.7)	102 (25.0)	0.928^b^
^†^Diabetes mellitus (*n*, %)	107 (45.5)	131 (32.1)	0.001^b^
^†^History of hypertension (*n*, %)	148 (63.0)	230 (56.4)	0.101^b^
^†^Family history (*n*, %)	37 (15.7)	54 (13.2)	0.379^b^
^&^Systolic blood pressure (mmHg)	134.4 ± 19.5	132.4 ± 17.1	0.173^a^
^&^Diastolic blood pressure (mmHg)	82.2 ± 34.0	78.6 ± 11.2	0.053^a^
Previous medications (*n*, %)			
^†^Aspirin (*n*, %)	130 (55.3)	215 (52.7)	0.521^b^
^†^Clopidogrel (*n*, %)	98 (41.7)	156 (38.2)	0.386^b^
^†^Statins (*n*, %)	112 (47.7)	204 (50.0)	0.568^b^
^†^ACEI/ARB (*n*, %)	61 (26.0)	104 (25.5)	0.896^b^
^†^*β*-blocker (*n*, %)	71 (30.2)	111 (27.2)	0.415^b^
^†^Calcium channel blocker (*n*, %)	59 (25.1)	99 (24.3)	0.811^b^

^&^Data are expressed as the mean ± SD. ^†^Data are expressed as *n* (%). ^a^*p*-value by independent sample *t*-test. ^b^*p*-value by *χ*^2^ test. CCC, coronary collateral circulation; BMI, body mass index; ACEI, angiotension converting enzyme inhibitors; ARB, angiotensin receptor blocker.

### Biochemical indicators in the poor and good collateral circulation groups

White blood cell count, absolute neutrophil count, platelet count, absolute monocyte count, red blood cell count, hemoglobin, red blood cell distribution width, fasting glucose, D-Dimer, fibrinogen, total cholesterol, low-density lipoprotein cholesterol, high-density lipoprotein cholesterol, triglycerides, creatinine, glomerular filtration rate, albumin, troponin and B-type natriuretic peptide levels were not significantly different between the two groups (all *P* > 0.05). The mean MPV of the poor CCC group (9.56 ± 1.49) was significantly higher than that of the good CCC group (8.52 ± 1.07, *P* < 0.01), while the mean lymphocyte count of the poor CCC group was significantly lower [(1.40 ± 0.50) × 10^9^/L] than that of the good CCC group [(1.89 ± 0.54) × 10^9^/L, *P* < 0.01]. The MPVLR of the poor CCC group (7.82 ± 3.80) was significantly higher than that of the good CCC group (4.84 ± 1.42, *P* < 0.01). Finally, the CRP of the poor CCC group was significantly higher than that of the good CCC group (*P* < 0.01, Table 2).

**TABLE 2:** Comparison of biochemical parameters between the two groups.

Variables	Poor CCC (*n* = 235)	Good CCC (*n* = 408)	*P*-value
^&^White blood cells/ (×10^9^/L)	6.59 ± 2.16	6.89 ± 1.78	0.054^a^
^&^Red blood cell/ (×10^12^/L)	4.36 ± 0.56	4.45 ± 0.53	0.057^a^
^&^Platelets (×10^9^/L)	211.03 ± 66.06	217.83 ± 52.89	0.153^a^
^&^Hemoglobin (g/L)	133.78 ± 17.64	136.37 ± 16.06	0.064^a^
^&^Absolute neutrophil count (×10^9^/L)	4.47 ± 1.92	4.37 ± 1.47	0.479^a^
^&^Absolute lymphocyte count (×10)^9^/L	1.40 ± 0.50	1.89 ± 0.54	<0.001^a^
^&^Monocytes (×10^9^/L)	0.48 ± 0.20	0.51 ± 0.18	0.080^a^
^&^Hematocrit (L/L)	0.40 ± 0.0496	0.41 ± 0.0462	0.056^a^
^&^Mean Corpuscular Volume (fL)	92.48 ± 4.87	92.96 ± 4.83	0.227^a^
^&^Red blood cell distribution width (%)	13.40 ± 1.03	13.35 ± 0.86	0.451^a^
^&^Platelet hematocrit (%)	0.19 ± 0.053	0.19 ± 0.048	0.347^a^
^&^Platelet distribution width (fL)	16.58 ± 0.55	16.51 ± 0.51	0.088^a^
^&^Mean Platelet Volume (fL)	9.56 ± 1.49	8.52 ± 1.07	<0.001^a^
^‡^Troponin I (ng/mL)	0.012 (0.010–0.030)	0.012 (0.010–0.040)	0.889^c^
^‡^B-type natriuretic peptide (pg/mL)	337.06 (116.0–844.0)	325.07 (137.5–676.5)	0.554^c^
^&^Creatinine (μmol/L)	75.62 ± 20.28	74.93 ± 18.77	0.661^a^
^&^Glomerular filtration rate (ml/min/1.73 m^2^)	89.09 ± 16.70	91.33 ± 16.95	0.106^a^
^&^Fasting blood glucose (mmol/L)	6.08 ± 2.10	5.82 ± 2.01	0.119^a^
^&^High density lipoprotein (mmol/L)	0.97 ± 0.20	0.93 ± 0.23	0.061^a^
^&^Low density lipoprotein (mmol/L)	2.16 ± 0.82	2.22 ± 0.81	0.369^a^
^&^Total cholesterol (mmol/L)	3.66 ± 0.96	3.67 ± 0.94	0.854^a^
^‡^Triglyceride (mmol/L)	1.38 (1.06–1.89)	3.10 (1.38–7.33)	0.496^c^
^&^Albumin (g/L)	41.31 ± 4.63	41.89 ± 3.42	0.068^a^
^&^D-Dimer (mg/L)	0.238 ± 0.056	0.240 ± 0.059	0.557^a^
^&^Fibrinogen (g/L)	3.01 ± 0.70	2.97 ± 0.63	0.412^a^
^&^Left ventricular ejection fraction (%)	58.32 ± 8.3	58.08 ± 8.3	0.715^a^
^‡^C-reactive protein (mg/L)	3.57 (1.50–8.65)	1.60 (0.90–4.088)	<0.001^c^
^&^Mean platelet volume-to-lymphocyte ratio	7.82 ± 3.80	4.84 ± 1.42	<0.001^a^

^&^Data are expressed as the mean ± SD. ^‡^Data are expressed as median (interquartile range). ^a^*p*-value by independent sample *t*-test. ^c^*p*-value by Mann–Whitney *U* test. CCC, coronary collateral circulation.

Spearman correlation analysis demonstrated that MPVLR was negatively correlated with CCC grade, and decreased with increasing Rentrop grade (*r* = –0.560, *P* < 0.01). The MPVLR of patients with Rentrop grade 0 (10.02 ± 4.50) was significantly higher than that of patients with Rentrop grade 1 (6.08 ± 1.73, *P* < 0.05) and the MPVLR of patients with Rentrop grade 1 (6.08 ± 1.73) was significantly higher than that of patients with Rentrop grade 2 (4.96 ± 1.35, *P* < 0.05), but the MPVLR of patients with Rentrop grade 2 (4.96 ± 1.35) was not significantly different than that of patients with Rentrop grade 3 (4.70 ± 1.49, *P* > 0.05).

### Coronary angiographic characteristics of patients with poor and good collateral circulation

The proportions of patients with multi-vessel coronary artery disease and right coronary artery occlusion in the good CCC group were significantly higher than those of the poor CCC group (both *P* < 0.01, Table 3).

**TABLE 3:** Comparison of coronary angiographic characteristics between the two groups.

	Poor CCC (*n* = 235)	Good CCC (*n* = 408)	*P*-value
**Occlusion of blood vessels**			
Left anterior descending branch	118 (50.2%)	175 (42.9%)	0.073
Left circumflex branch	63 (26.8%)	129 (31.6%)	0.199
Right coronary artery	71 (30.2%)	235 (57.6%)	<0.001
**Number of diseased vessels**			
One-vessel disease	52 (22.1%).	54 (13.2%).	0.003
Two-vessel disease	91 (38.7%)	134 (32.8%)	0.132
Three-vessel disease	92 (39.1%)	220 (53.9%)	<0.01
**Rentrop collateral grading**			
0	104 (44.3%)		
1	131 (55.7%)		
2		218 (53.4%)	
3		190 (46.6%)	
Multivessel lesions	183 (77.9%)	354 (86.8%)	0.003

Data are expressed as *n* (%), *p*-value by *χ*^2^ test. CCC, coronary collateral circulation.

### Multivariate analysis of factors related to coronary collateral circulation formation

With Rentrop classification group as the dependent variable and factors with statistical significance (*P* < 0.05) in univariate comparisons as the independent variables, MPVLR (OR = 2.101, *P* < 0.01), diabetes (OR = 2.355, *P* < 0.01), C-reactive protein level (OR = 1.036, *P* < 0.05), and right coronary artery occlusion (OR = 0.313, *P* < 0.01) were independently related to CCC formation (Table 4).

**TABLE 4:** Univariate and multivariate logistic regression analyses of poor coronary collateral circulation in CTO patients.

Variables	Univariate analysis			Multivariate analysis		
		
	b	Odds ratio, 95% CI	*P*-value	b	Odds ratio, 95% CI	*P*-value
Diabetes mellitus	0.570	1.768 (1.270–2.459)	0.001	0.857	2.355 (1.532–3.621)	< 0.001
RCA occlusion	–1.143	0.319 (0.227–0.448)	< 0.001	–1.161	0.313 (0.202–0.485)	< 0.001
MPVLR	0.695	2.004 (1.775–2.262)	< 0.001	0.742	2.101 (1.840–2.399)	< 0.001
C-reactive protein	0.039	1.040 (1.016–1.064)	0.001	0.035	1.036 (1.008–1.064)	0.011
Multivessel lesions	–0.644	0.525 (0.344–0.801)	0.003	–0.425	0.654 (0.374–1.144)	0.137

CTO, chronic total occlusion; RCA, right coronary artery; MPVLR, mean platelet volume-to-lymphocyte ratio; CI, confidence interval.

### Receiver operating curve analysis

**FIGURE 1:**
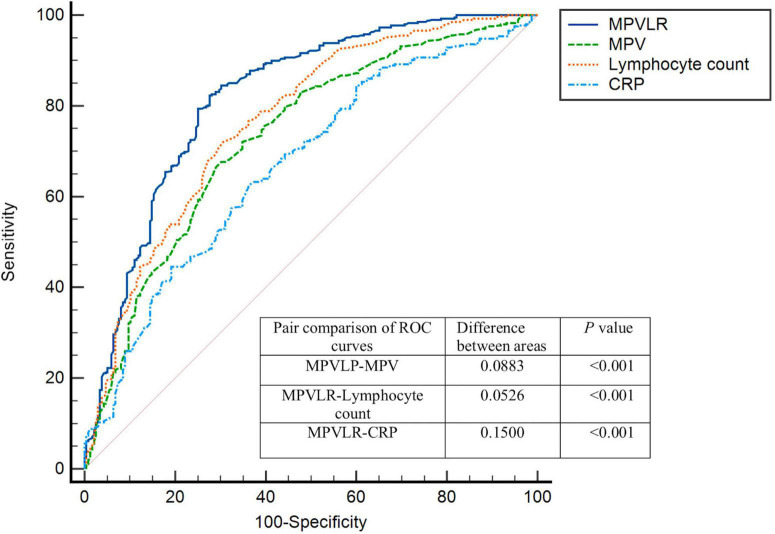
Receiver-operating characteristics (ROC) curves for MPVLR, MPV, CRP, and lymphocyte count value in the prediction of impaired coronary collateral circulation. MPVLR, mean platelet volume to lymphocyte ratio; MPV, mean platelet volume; CRP, C-reactive protein.

The Table 5 showed the ROC curve parameters of MPVLR, MPV, lymphocyte count, and CRP for predicting CCC formation, with the area under the curve (AUC) of 0.820, 0.731, 0.767, and 0.670, respectively (all *p* < 0.01); The AUC of MPVLR was compared with that of MPV, lymphocyte count and CRP. All comparisons had *P*-values < 0.01, indicating that the AUC of MPVLR was significantly different from that of MPV, lymphocyte count and CRP (Figure 1). The four variables (X1: MPVLR; X2: lymphocyte count; X3: MPV; X4: CRP) and their regression coefficients were used to establish the regression equation: Y (the probability of poorly formed CCC) = 1/[1 + e^–(–8.025 + 0.731 * X1+0.442 * X2 + 0.254 * X3 + 0.033 * X4)^]. The Omnibus test of the regression equation (*χ*^2^ = 244.843, *P* < 0.001) showed the regression equation was statistically significant. Hosmer Lemeshow Test showed that the regression equation had a good fitting degree (*χ*^2^ = 9.541, *P* = 0.299). The above results showed that the predictive value of MPVLR for poor CCC in CTO patients was better than that of MPV, CRP, and lymphocyte count.

**TABLE 5:** Receiver-operating characteristic (ROC) curve parameters of MPVLR, MPV, lymphocyte count, and CRP for predicting CCC formation.

Variable	Cutoff point	AUC	95% CI	Sensitivity	Specificity	*P*-value
MPVLR	6.02	0.820	0.784–0.855	72.3%	82.4%	<0.001
Lymphocyte count	1.57	0.767	0.728–0.806	72.1%	69.4%	<0.001
MPV	8.70	0.731	0.691–0.772	71.1%	66.4%	<0.001
CRP	2.29	0.670	0.627–0.713	63.8%	62.5%	<0.001

ROC, receiver-operating characteristics; MPVLR, mean platelet volume to lymphocyte ratio; MPV, mean platelet volume; CRP: c-reactive protein; CCC, coronary collateral circulation; AUC, area under the curve.

## Discussion

Coronary artery collateral vessel formation is a complex process that includes both angiogenesis and arteriogenesis (18). Angiogenesis is a complex and orderly process that involves a variety of growth factors and adhesion molecules that are secreted and expressed by a variety of different types of cells (such as endothelial cells and smooth muscle cells), in particular endothelial cells (19–22). Chronic inflammation can lead to endothelial dysfunction in a variety of ways, most prominently through increased production of reactive oxygen species (23), which affects the formation of CCC. Lymphocytes are important to innate immunity. Regulatory T cells in particular have significant anti-inflammatory effects. Lymphocyte counts can be reduced in response to inflammation, possibly by increased steroid levels due to stress or apoptosis (24). This decrease in lymphocytes, especially T lymphocytes, leads to decreased vascular infiltration and reductions in vascular endothelial growth factor (VEGF) and other factors related to collateral angiogenesis, thereby inhibiting CCC production (25). Mean platelet volume represents the average volume of a single platelet. It can not only reflect the proliferation and metabolism of megakaryocytes in the bone marrow and platelet production, but also the life span of platelets in the peripheral circulation (26). Large platelets contain more vasoactive substances and prothrombotic factors, such as platelet factor 4, P-selectin, platelet-derived growth factor, dense particles and thromboxane A2, which can regulate the inflammatory response and endothelial permeability and thereby inhibit the formation of CCC. The higher the MPV, the more active the metabolic response and the faster the thrombosis and inflammation process (27). Hadadi et al. (10) showed that MPV and platelet-to-lymphocyte ratio can predict the presence of CTO in patients presenting with STEMI.

Mean platelet volume lymphocyte ratio is an inflammatory marker that combines mean platelet volume and lymphocyte count. MPVLR is therefore more stable and objective than a single index, and can reflect both the inflammatory response and thrombosis levels. Ornek et al. (13) found that MPVLR is associated with coronary collateral circulation formation in stable angina patients. However, these reports did not utilize a Chinese population. This validative study of 643 patients with CTO found that the MPVLR level in CTO patients decreased with increased coronary collateral grade, and a high MPVLR level could independently predict poor CCC formation in CTO patients. The ROC curve showed that the AUC of MPVLR in the prediction of poor CCC formation was 0.820. The optimal cut-off point was 6.02, with a sensitivity of 72.3% and a specificity of 82.4%. These results suggest that MPVLR, a simple, feasible and inexpensive non-invasive biomarker, may be a clinical predictor of poor CCC in CTO patients.

C-reactive protein, right coronary CTO and diabetes mellitus were independent risk factors for poor coronary collateral circulation. Vascular endothelial cells and nitric oxide (NO) play a crucial role in the formation of coronary collateral circulation. NO not only regulates the functional activity of CCC by relaxing small vessels, but also mediates the angiogenesis of VEGF (28). Fan et al. (29) showed that CRP was significantly associated with poor CCC formation, and therefore could be used as an independent predictor of poor CCC. The results of our study showed that the CRP levels of patients with poor CCC formation were higher than in those with good CCC formation, and high CRP level was negatively correlated with CCC formation in CTO patients. The mechanism behind CRP inhibition may be that CRP inhibits the biological activity and expression of endothelial nitric oxide synthase in endothelial progenitor cells, thereby inhibiting the synthesis of NO and leading to endothelial dysfunction (30, 31). CRP can also inhibit VEGF-induced endothelial cell migration (32). Both CRP and MPVLR, as inflammatory markers, are associated with poor CCC formation. It may therefore be possible to improve the condition and prognosis of CTO patients by reducing the inflammatory response and promoting CCC formation.

Similar to prior work (33), the present study showed that right coronary artery occlusion was more likely to form better collateral circulation than left anterior descending and left circumflex artery occlusion. The mechanism behind this may be related to the formation of collateral circulation and the increase of shear stress. The pressure differential caused by vascular occlusion increased fluid shear stress in arterioles, which leads to upregulation of adhesion molecules in endothelial cells and VEGF production by activated monocytes. At the same time, shear stress stimulates endothelial cells to produce basic fibroblast growth factor (bFGF) and platelet-derived growth factor (PDGF), leading to the mitosis of endothelial and smooth muscle cells. Shear stress may also directly stimulate the production of growth factors such as PDGF, bFGF, and transforming growth factor *β* in endothelial cells (20). The association between right coronary artery occlusion and good collateral circulation formation may be due to the higher driving pressure of the right coronary artery and lower right ventricular tension during ventricular systole, which results in a larger coronary pressure gradient and promotes the development of collateral circulation (34).

The results of this study also showed that the proportion of diabetic patients with poor CCC was higher than that of patients with good CCC, suggesting that diabetes is related to poor CCC establishment and opening. Elevated blood glucose level and insulin resistance can lead to endothelial cell dysfunction, resulting in reduced NO and pro-angiogenic factor secretion and the inhibition of pro-angiogenic factor activity (35, 36). Blood glucose control and reduced insulin resistance in diabetic patients may avoid reduced collateral circulation in these patients. Our study showed that multivascular lesions was more likely to form better collateral circulation than single vessel disease. The mechanism may be that CCC is caused by remodeling process (arteriogenesis) which is triggered by shear stress acting on endothelial cells, and the sprouting of new vessels (angiogenesis) induced by ischemic tissue (37). In the multivascular lesions patients, the severity of ischemia and coronary stenosis was significantly more serious than single vessel disease patients, thus promoting a better collateral circulation formation.

### Study limitations

Our study has several limitations. CCC was assessed using coronary angiography alone, without the use of intravascular ultrasound. Second, this was a single-center retrospective study, and further large sample studies are needed to confirm our results.

## Conclusion

In conclusion, elevated MPVLR level was independently associated with CCC dysplasia in patients with coronary CTO. MPVLR level may therefore be helpful in the prediction of CCC formation in patients with coronary CTO.

## Data availability statement

The raw data supporting the conclusions of this article will be made available by the authors, without undue reservation.

## Ethics statement

The studies involving human participants were reviewed and approved by the Ethics Committee of the First Affiliated Hospital of Zhengzhou University. The patients/participants provided their written informed consent to participate in this study.

## Author contributions

FH and M-HN conceived and designed the study. M-HN performed the statistical analysis. M-HN and RG interpreted results. M-HN, FH, and RG drafted the report. RG, P-HL, Z-HL, and J-WZ provided critical suggestions for improving the manuscript. All authors contributed to data acquisition and to the article and approved the submitted version.
